# Rich Oxygen Vacancies Induced by Surface Self-Reconstruction in Sandwich-like Hierarchical Structured Electrocatalyst for Boosting Oxygen Evolution Reaction

**DOI:** 10.3390/molecules30122632

**Published:** 2025-06-17

**Authors:** Xiaoguang San, Wanmeng Wu, Xueying Li, Lei Zhang, Jian Qi, Dan Meng

**Affiliations:** 1 College of Chemical Engineering, Shenyang University of Chemical Technology, Shenyang 110142, China; sanxiaoguang@syuct.edu.cn (X.S.); 2022027@stu.syuct.edu.cn (W.W.); l15940542952@163.com (X.L.); 2 State Key Laboratory of Biochemical Engineering, Institute of Process Engineering, Chinese Academy of Sciences, Beijing 100190, China; 3 School of Chemical Engineering, University of Chinese Academy of Sciences, Beijing 100049, China

**Keywords:** oxygen evolution reaction, nickel–iron layered double hydroxide, oxygen vacancies, self-reconstruction

## Abstract

The oxygen evolution reaction (OER) is pivotal in hydrogen production via water electrolysis, yet its sluggish kinetics, stemming from the four-electron transfer process, remain a major obstacle, with overpotential reduction being critical for enhancing efficiency. This work addresses this challenge by developing a novel approach to stabilize and activate non-precious metal catalysts for OER. Specifically, we synthesized a three-dimensional flake NiFe-LDH/ZIF-L composite catalyst on a flexible nickel foam (NF) substrate through a room temperature soaking and hydrothermal method, leveraging the mesoporous structure of ZIF-L to increase the specific surface area and optimizing electron transfer pathways via interfacial regulation. Continuous linear sweep voltammetry (LSV) scanning induced structural self-reconstruction, forming highly active NiOOH species enriched with oxygen vacancies, which significantly boosted catalytic performance. Experimental results demonstrate an overpotential of only 221 mV at 10 mA cm^−2^ and a Tafel slope of 56.3 mV dec^−1^, alongside remarkable stability, attributed to the catalyst’s hierarchical nanostructure that accelerates mass diffusion and charge transfer. The innovation lies in the synergistic effect of the mesoporous ZIF-L structure and interfacial regulation, which collectively enhance the catalyst’s activity and durability, offering a promising strategy for advancing large-scale water electrolysis hydrogen production technology.

## 1. Introduction

Hydrogen energy, with its high calorific value, cleanliness, zero pollution, and wide range of raw materials and applications, is hailed as the “ultimate energy” of the 21st century [1,2,3]. Currently, the widespread use of “gray hydrogen” produced from fossil fuels such as natural gas and coal exacerbates carbon emissions. As a result, water electrolysis for hydrogen production has emerged as a promising renewable energy-based hydrogen production technology [4]. By using water as a feedstock, it avoids the use of fossil fuels and can be integrated with renewable energy sources such as solar and wind power for sustainable production [5,6,7,8]. The oxygen evolution reaction (OER), involving a four-electron transfer proton-coupled process, is kinetically slow and represents the rate-determining step in water electrolysis. Current research on OER catalysts focuses on enhancing catalytic efficiency, reducing costs, and improving stability [9,10,11]. Commercial oxygen evolution catalysts are mainly precious metal oxide catalysts such as IrO_2_ and RuO_2_, which are scarce and expensive, greatly limiting their large-scale industrial application. Transition metal-based catalysts, especially nickel-based ones, are widely used due to their low cost and abundant Earth reserves [12,13,14,15]. Nickel–iron layered double hydroxide (NiFe-LDH) is one of the most studied electrocatalysts for water electrolysis. Among them, Ni and Fe promote efficient electron transport between active sites through valence state regulation and synergistic changes in lattice geometry, enhance the adsorption and activation of reactants and intermediates, and thus significantly improve the catalytic performance of the oxygen evolution reaction [16,17]. In fact, NiFe-LDH acts as a precursor catalyst, undergoing significant self-reconstruction in alkaline solutions to form nickel oxide (NiOOH) as the active species for OER [18,19,20]. Metal–organic frameworks (MOFs) have also attracted considerable attention because they undergo a phase transition during the alkaline electrolysis process, referred to as “self-structural reconstruction.” During this process, the organic ligands in MOFs are uncontrollably replaced by hydroxide ions, resulting in the formation of metal hydroxides [21,22,23].

The primary approach to enhance the electrocatalytic activity of OER catalysts involves modulating their electronic structure and microstructure. Defect engineering, doping, and interface regulation are methods employed to optimize the electronic structure of catalysts, thereby enhancing their catalytic performance [24,25]. The defect engineering strategy can promote the reconstruction of MOFs, creating abundant oxygen vacancies in metal hydroxide oxides, which are beneficial for the adsorption of oxygen-containing intermediates at the active sites, significantly improving the OER performance [25,26,27]. The bonding and charge redistribution induced by metal dopants in NiO facilitate surface reconstruction, leading to the generation of surfaces enriched with Ni^3+^ and oxygen vacancies (O_v_), thus increasing the number of active sites and enhancing the intrinsic catalytic activity [28]. At the same time, the establishment of heterogeneous interfaces can trigger electron rearrangement, promoting the transition of electrons from the t_2g_ orbitals to the e_g_ orbitals, achieving a half-filled state, which optimizes the adsorption of oxygen intermediates on the M sites (M^3+^/M^4+^) [29]. Designing the microstructure of catalysts is also an effective approach to enhance catalytic performance. Crystal morphology and dispersion are key factors influencing the catalytic performance of LDH. Controlling the morphology of catalysts to achieve monodisperse two-dimensional structures, such as nanosheets or nanoflowers, not only increases the exposed active sites and improves the apparent activity but also accelerates gas desorption, thereby improving material diffusion [30,31,32]. Furthermore, rationally tuning the exposed high-activity crystal facets can enhance the quality-to-activity ratio of the nickel-based catalyst in OER, lower the reaction energy barrier, and increase the catalytic reaction rate [33].

Herein, flake-like zeolitic imidazolate framework (ZIF-L) arrays with smooth surfaces are fabricated in situ on flexible nickel foam (NF) substrates via a soaking method at room temperature. Subsequently, a sandwich-like sheet composite was obtained by the hydrothermal growth of NiFe-LDH on ZIF-L/NF. The hierarchical nanostructure of the catalyst ensured that the active sites were fully exposed to the electrolyte. Additionally, the interface interaction between ZIF-L and NiFe-LDH will accelerate charge transfer. Continuous LSV scanning induced the reconstruction of NiFe-LDH/ZIF-L/NF, generating abundant oxygen vacancies and highly active intermediates that enhance OER performance. As a result, a low overpotential of 221 mV was achieved to reach 10 mA cm^−2^, with good stability.

## 2. Results and Discussion

**Figure 1:**
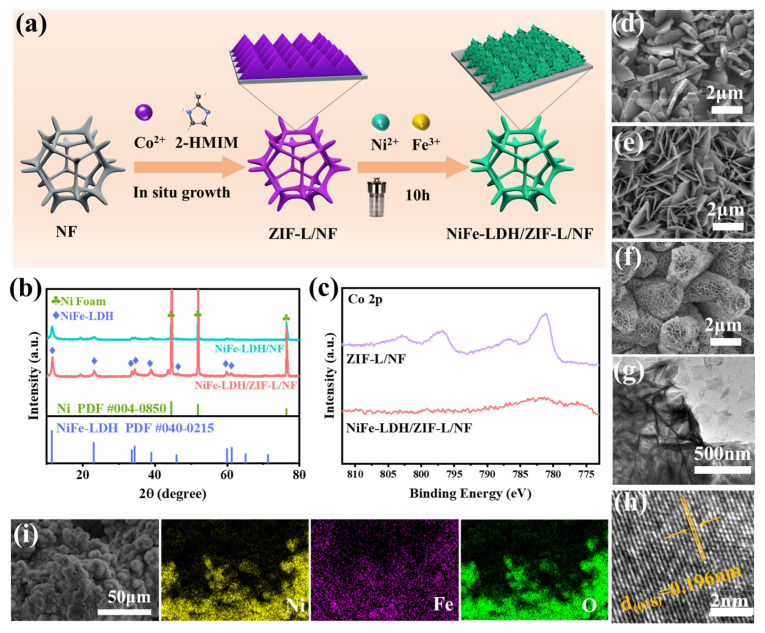
(**a**) Illustration of synthesis of NiFe LDH/ZIF-L/NF. (**b**) XRD patterns of NiFe-LDH/NF and NiFe-LDH/ZIF-L/NF. (**c**) Co 2p XPS spectra of ZIF-L/NF and NiFe-LDH/ZIF-L/NF. SEM images of (**d**) ZIF-L/NF, (**e**) NiFe-LDH/NF, and (**f**) NiFe-LDH/ZIF-L/NF. (**g**) TEM image, (**h**) HR-TEM image, and (**i**) EDS patterns of NiFe-LDH/ZIF-L/NF.

Figure 1a shows the preparation process of the sandwich composite electrocatalyst (NiFe-LDH/ZIF-L/NF). In order to avoid the influence of the nickel foam substrate on the analysis results, we peeled ZIF-L from the nickel foam substrate and performed X-ray diffraction (XRD) tests. The sample showed obvious diffraction peaks at 10.4°, 15.2°, and 18.1°, which corresponded to the (002), (022), and (222) crystal planes of the cubic phase ZIF-L, respectively, which were consistent with existing literature reports (Figure S1) [34], proving the successful synthesis of ZIF-L/NF. In addition, we also performed Fourier transform infrared spectroscopy (FTIR) analysis on it, and all absorption peaks were consistent with existing literature results (Figure S2) [35], further proving the successful synthesis of ZIF-L/NF composite materials. NiFe-LDH was in situ-grown on ZIF-L/NF using a hydrothermal method. In Figure 1b, the diffraction peaks at 11.4°, 22.9°, 33.5°, 34.4°, 38.9°, 45.9°, 59.9°, and 61.2° corresponded to the (003), (006), (101), (012), (015), (018), (110), and (113) crystal planes in the standard card of NiFe-LDH (PDF#040-0215), respectively, proving that NiFe-LDH was successfully grown on ZIF-L/NF [36]. Compared with NiFe-LDH/NF, the diffraction peak intensity of NiFe-LDH/ZIF-L/NF is higher, indicating that ZIF-L-modified nickel foam is more conducive to the growth of NiFe-LDH. Further, through X-ray photoelectron spectroscopy (XPS) analysis, we compared the Co 2p spectra of ZIF-L/NF and NiFe-LDH/ZIF-L/NF. The results showed that no Co signal was detected in the NiFe-LDH/ZIF-L/NF sample (Figure 1c), which further proved that NiFe-LDH was uniformly coated on ZIF-L to form a sandwich structure. The morphology of the catalyst was characterized by scanning electron microscopy. ZIF-L grows uniformly on the three-dimensional NF skeleton, showing a leaf-shaped nanosheet array, while NiFe-LDH is a thinner two-dimensional cross-linked nanosheet (Figure 1d,e). It can be clearly seen that NiFe-LDH grows uniformly on the ZIF-L/NF surface, and NiFe-LDH shows thin nanosheets with smaller sizes, forming a sandwich composite sheet structure (Figure 1f,g). This special structure will solve the problem of poor conductivity and agglomeration of LDHs, with a significant number of Ni and Fe sites at the edges serving as the main active sites for OER, thus enhancing catalytic performance. The high-resolution TEM (HRTEM) of NiFe-LDH/ZIF-L/NF revealed clear lattice fringes, with a spacing of 0.196 nm corresponding to the (018) crystal plane of NiFe-LDH (Figure 1h). The EDS pattern and XPS spectra show O, Ni, and Fe on the NiFe-LDH/ZIF-L/NF surface (Figure 1i and Figure S3).

**Figure 2:**
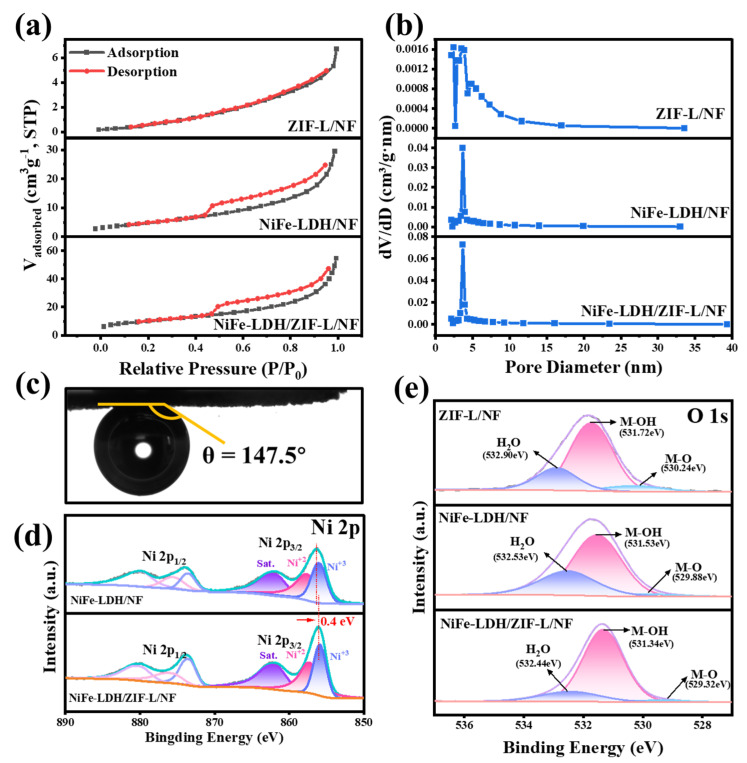
(**a**) N_2_ adsorption–desorption isotherm and (**b**) pore size distribution of ZIF-L/NF, NiFe-LDH/NF, and NiFe-LDH/ZIF-L/NF. (**c**) Gas contact angles of NiFe-LDH/ZIF-L/NF. (**d**) Ni 2p XPS of NiFe-LDH/NF and NiFe-LDH/ZIF-L/NF, and (**e**) O 1s XPS of ZIF-L/NF, NiFe-LDH/NF, and NiFe-LDH/ZIF-L/NF.

Nitrogen adsorption–desorption isotherms indicate that all samples exhibit the characteristics of type IV adsorption isotherms, and pore size distribution analysis confirms the mesoporous nature of these samples (Figure 2a,b), which will play a crucial role in enhancing the mass transfer of reactants and increasing the exposure of active sites [37,38]. Compared to ZIF-L/NF and NiFe-LDH/NF, the BET specific surface area of NiFe-LDH/ZIF-L/NF is significantly increased. However, its pore size distribution becomes smaller due to the uniform in situ growth of NiFe-LDH on the surface of ZIF-L (Table S1), resulting in an increase in pore volume and the formation of a looser and more porous structure (Figure S4). This allows for more effective contact between the electrolyte and catalyst surface, will facilitate rapid material diffusion, and benefit mass transfer during the OER process. Dynamic contact angle observations of NiFe-LDH/ZIF-L/NF (Video S1) reveal that water droplets immediately disappear upon contact with the sample surface, demonstrating superhydrophilicity [39]. This is attributed to the catalyst’s porous structure and the presence of abundant hydrophilic groups. The underwater aerophobic property of the electrode surface is crucial for its OER catalytic performance. NiFe-LDH/ZIF-L/NF has a gas contact angle of 147.5° (Figure 2c) and has good gas transport on the surface, which facilitates bubble separation and accelerates reaction kinetics [39,40,41]. In Figure 2d, the two peaks of NiFe-LDH/NF at binding energies of 856.5 eV and 874.2 eV are Ni 2p_3/2_ and Ni 2p_1/2_, respectively, where Ni 2p_3/2_ contains two material forms, Ni^3+^ and Ni^2+^. Compared with Ni 2p of NiFe-LDH/NF, NiFe-LDH/ZIF-L/NF shifts 0.4 eV toward the low-binding-energy direction, which is attributed to the interfacial interaction between NiFe-LDH and ZIF-L, which will facilitate rapid electron transfer and accelerate the OER kinetics [42]. In Figure 2e, for the XPS spectra of O 1s, ZIF-L/NF, NiFe-LDH/NF, and NiFe-LDH/ZIF-L/NF can all be deconvoluted into three peaks, with the peaks of 532.4~532.9 eV being metal oxides (M–O), 531.3~531.7 eV being metal hydroxyls (M–OH), and 529.3~530.3 eV being surface-adsorbed water (H_2_O). Interestingly, NiFe-LDH/ZIF-L/NF shows a slight shift towards lower binding energies compared to ZIF-L/NF and NiFe-LDH/NF, further confirming charge transfer between NiFe-LDH and ZIF-L [42,43,44]. Therefore, the interfacial interaction between NiFe-LDH and ZIF-L can alter the chemical state and binding energy of O elements, which is beneficial for improving OER activity.

**Figure 3:**
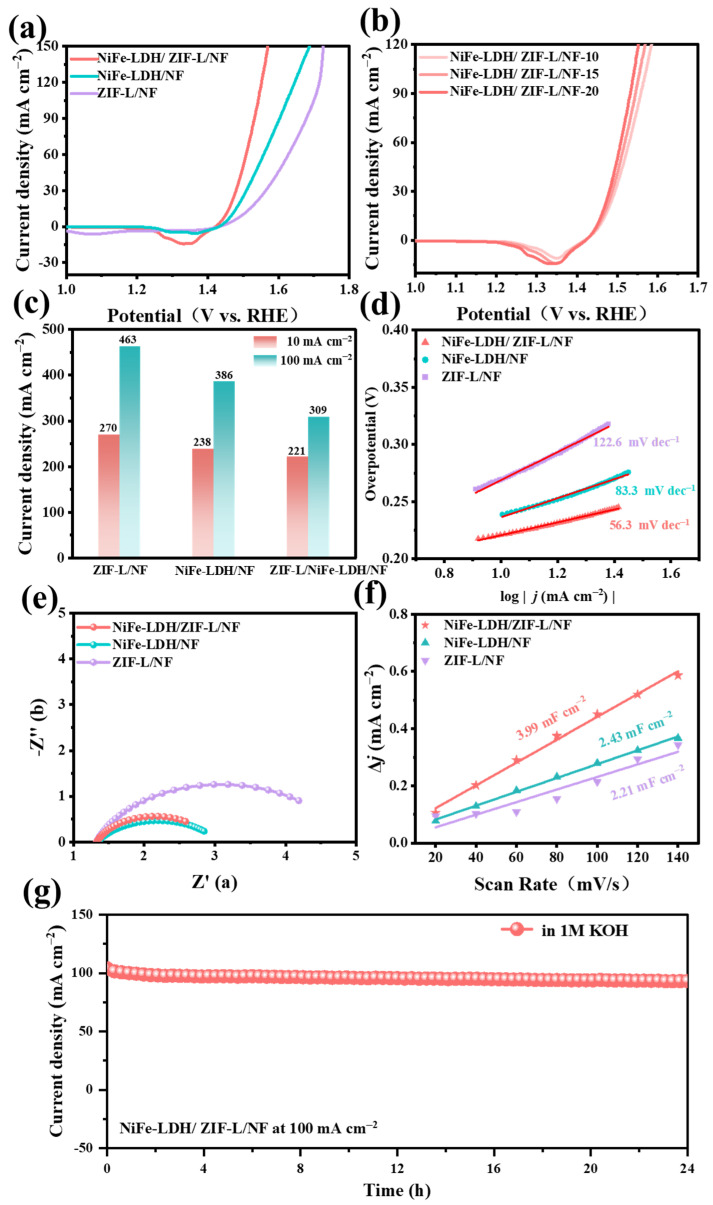
LSV curves of (**a**) the as-prepared catalysts for the OER in 1.0 M KOH, (**b**) NiFe-LDH/ZIF-L/NF in different cycles, and (**c**) the overpotential at 10 and 100 mA cm^−2^; (**d**) the corresponding Tafel slope derived from the LSV curves, (**e**) the EIS curve of potential at 10 mA cm^−2^, (**f**) the corresponding Cdl value, and (**g**) chronopotentiometry test of the catalyst at a constant potential of 1.54 V vs. RHE in 1.0 M KOH electrolyte for 24 h to evaluate the electrochemical stability of NiFe-LDH/ZIF-L/NF.

In order to achieve structural self-reconstruction, NiFe-LDH/ZIF-L/NF was subjected to continuous linear sweep voltammetry (LSV) scanning in potassium hydroxide solution without activation. This process induced the formation of a highly disordered Co(OH)_2_ structure inside ZIF-L, which would trigger the conversion of NiFe-LDH into intermediates with higher OER activity, thereby enhancing the intrinsic activity of OER. As the number of LSV curves increased to 20, NiFe-LDH/ZIF-L/NF exhibited excellent catalytic performance, with a much lower overpotential than comparative catalysts under the same conditions. The overpotentials were 221/309 mV for NiFe-LDH/ZIF-L/NF, 238/386 mV for NiFe-LDH/NF, and 270/463 mV for ZIF-L/NF at 10 mA cm^−2^ and 100 mA cm^−2^, respectively (Figure 3a–c). NiFe-LDH/ZIF-L/NF had the smallest Tafel slope of only 56.3 mV dec^−1^, compared to NiFe-LDH/NF (83.3 mV dec^−1^) and ZIF-L/NF (122.6 mV dec^−1^), indicating rapid reaction kinetics for OER (Figure 3d). Electrochemical impedance spectroscopy (EIS) analysis (Figure 3e) showed that the charge transfer resistance (RCT) of NiFe-LDH/ZIF-L/NF (1.64 Ω) was also smaller than that of NiFe-LDH/NF (1.73 Ω) and ZIF-L/NF (3.94 Ω), demonstrating its good conductivity. In addition, the corresponding electrochemical double layer capacitance (Cdl) was obtained by cyclic voltammetry (CV) measurement (Figure S5), and it is generally believed that the electrochemical active surface area (ECSA) is proportional to its Cdl value. As shown in Figure 3f, NiFe-LDH/ZIF-L/NF shows the largest Cdl value (3.99 mF cm^−2^), and the ECSA of the catalyst (Table S2) is obtained by calculation. NiFe-LDH/ZIF-L/NF has the largest ECSA (4.32 m^2^/g), which means that it has a large active surface area, which is consistent with the BET results and is conducive to charge transport and ion dispersion. Compared with ZIF-L/NF and NiFe-LDH/NF, the LSV curve and Tafel slope have been normalized by the corresponding ECSA (Figure S6). NiFe-LDH/ZIF-L/NF still performs well, indicating that it has excellent intrinsic catalytic activity. Additionally, a long-term stability test of NiFe-LDH/ZIF-L/NF was conducted at 1.54 V to achieve 100 mA cm^−2^, confirming its satisfactory long-term stability (Figure 3g).

The material surface was reconstructed through continuous LSV scanning, 2-HMIM was almost completely dissolved, the coordination between metal ions and organic ligands was destroyed, a large number of unsaturated sites were generated, and the external force of the electric field reduced the energy barrier of surface reconstruction, which was conducive to the surface reconstruction process (Figure S7). Then, a series of characterizations were used to further study the morphology and structural evolution of the catalyst after reconstruction. In order to distinguish it from NiFe-LDH/ZIF-L/NF before the OER, we named the catalyst after the reaction NiFe-LDH/ZIF-L/NF-A.

**Figure 4:**
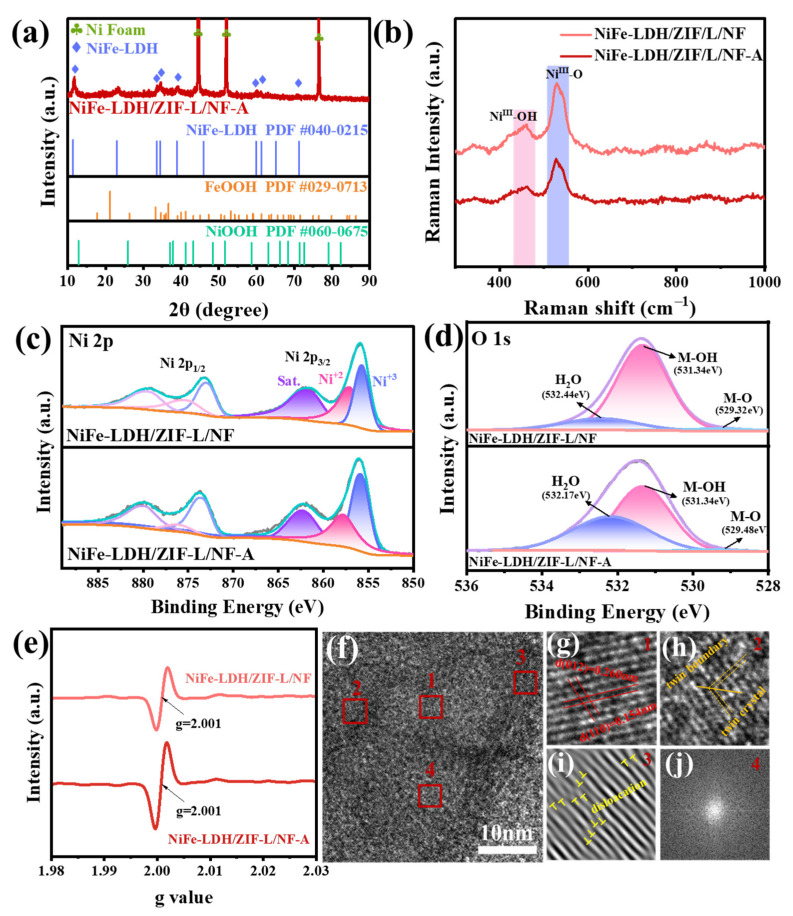
(**a**) XRD of NiFe-LDH/ZIF-L/NF-A.NiFe-LDH/ZIF-L/NF and NiFe-LDH/ZIF-L/NF-A: (**b**) Raman spectra, (**c**) Ni 2p XPS spectra, (**d**) O 1s XPS spectra, and (**e**) EPR spectra. (**f**) HRTEM image, (**g**) lattice fringes, (**h**) twin crystal, (**i**) dislocation, and (**j**) FFT mapping of NiFe-LDH/ZIF-L/NF-A.

In Figure 4a, the XRD peak of NiFe-LDH continues to exist, but the intensity of the diffraction peak is reduced compared to that before the reaction. There are also FeOOH and NiOOH diffraction peaks, which proves that NiFe-LDH transforms into hydroxyl oxide during the OER process [45]. In the Raman spectrum of NiFe-LDH/ZIF-L/NF-A (Figure 4b), two peaks appeared at 458 cm^−1^ and 526 cm^−1^, which were attributed to the bending vibration and stretching vibration of Ni^III^-O and Ni^III^-OH in NiFeOOH, respectively. After the reaction test, no new characteristic peaks appeared and there was no change, indicating that NiFe-LDH has good stability [46]. As the reaction proceeds, the morphology of NiFe-LDH/ZIF-L/NF changes significantly, showing a flower-like nanosheet structure. This transformation is attributed to the transformation process of ZIF-L to Co(OH)_2_. At the same time, the open nanostructure is conducive to the rapid diffusion of reactants and the efficient transfer of electrons, thereby improving the catalytic activity of the oxygen evolution reaction (OER). The element mapping results show that Ni, Fe, and O remain uniformly distributed in NiFe-LDH/ZIF-L/NF-A after the reaction, but a small amount of Co is exposed due to reconstruction (Figure S8). In Figure 4c, the binding energy of Ni 2p remains unchanged, though there is an increase in Ni^3+^ content (Table S3), indicating the formation of NiOOH throughout the OER, which is crucial for rapid reaction kinetics. The O 1s spectrums (Figure 4d) show an increase in adsorbed water, which can be explained by the generation of many water molecules during the reaction. Additionally, the M-OH/M-O ratio in the NiFe-LDH/ZIF-L/NF-A spectrum is higher than that in NiFe-LDH/ZIF-L/NF (Table S4), suggesting an increase in the number of oxygen vacancies during the LSV oxidation process, as the relative peak area ratio of M-OH/M-O can be used to roughly estimate the number of oxygen defects [47]. By analyzing the EPR spectra, the defect state of the catalyst was further detected. The appearance of the EPR signal (g value is 2.001) indicates that oxygen vacancies exist in all materials, and the number is different. In Figure 4e, the peak intensity of the reconstructed NiFe-LDH/ZIF-L/NF-A is greater than that of NiFe-LDH/ZIF-L/NF, which is consistent with the XPS results [48]. TEM images indicate that the catalyst retained its nanosheet morphology after the reaction, showing that the structure of NiFe-LDH was not significantly affected (Figure S9). Moreover, Figure 4f shows an HRTEM image of NiFe-LDH/ZIF-L/NF-A, where the magnified image of red area 1 (Figure 4g) shows that the lattice fringes with a plane spacing of 0.260 nm and 0.154 nm correspond to the (012) and (110) crystal planes of NiFe-LDH, respectively. Additionally, the magnified images of red regions 2–3 (Figure 4h,i) reveal the presence of numerous twin structures and edge dislocations within NiFe-LDH/ZIF-L/NF-A. Furthermore, the nanosheets of NiFe-LDH/ZIF-L/NF-A consist of both crystalline and amorphous regions [43]. A distinct amorphous structure is observed in red region 4, corresponding to Figure 4j, validated by the corresponding fast Fourier transform (FFT) mapping, suggesting that additional amorphous NiOOH may have formed during the reaction [49]. The results show that the reconstructed NiFe-LDH/ZIF-L/NF exhibits good catalytic performance and durability. Compared with the catalysts reported in the recent literature, it has excellent electrochemical performance (Table S5), and therefore it has the potential for practical application.

## 3. Experimental Setup

### 3.1. Materials

The reagents were supplied by the supplier without purification. Urea (CH_4_N_2_O), potassium hydroxide (KOH), cobaltous nitrate hexahydrate, nickel (II) nitrate hexahydrate (Ni(NO_3_)_2_·6H_2_O), (Co(NO_3_)_2_·6H_2_O), 2-Methylimidazole(C_4_H_6_N_2_), ferric nitrate nonahydrate (Fe(NO_3_)_3_·9H_2_O), and ammonium fluoride (NH_4_F) were sourced from Shanghai Macklin (Shanghai, China). Anhydrous ethanol (C_2_H_5_OH) was directly used as a commercial reagent from Tianjin Damao Chemical Reagent Factory (Tianjin, China). NF (NF is nickel foam) was bought from Suzhou Sinero Technology Co., Ltd. (Suzhou, China) Deionized (DI) water served as the universal solvent for all steps of the investigation.

ZIF/NF: First, the nickel foam with a thickness of 1 nm (1 × 2 cm^2^) was ultrasonically treated in 1.0 M HCl (to remove the impurities on the surface), DI water, and ethanol for 15 min and then dried. Amounts of 0.582 mg of Co(NO_3_)_2_·6H_2_O and 1.313 mg of 2-HM1M were weighed, added to 40 mL deionized water, and mixed thoroughly (Solution A). The pretreated nickel foam was added to Solution A and allowed to stand for 8 h. The product was washed with water and ethanol more than five times and dried in a vacuum at 60 °C for 12 h to obtain ZIF/NF.

NiFe-LDH/NF: Amounts of 0.262 mg of Ni(NO_3_)_2_·6H_2_O, 0.036 mg of Fe(NO_3_)_3_·9H_2_O, 0.118 mg of NH_4_F, and 0.480 mg of CO(NH_2_)_2_ were weighed, added to 20 mL of deionized water, and mixed by ultrasound (Solution B). Solution B and pretreated nickel foam (1 × 2 cm^2^) were transferred to a 25 mL PTFE reactor, heated at 120 °C for 10 h, cooled to room temperature, washed with water and ethanol more than five times, and vacuum-dried at 60 °C for 12 h to obtain NiFe-LDH/NF.

NiFe-LDH/ZIF-L/NF: ZIF-L/NF was added to solution B and transferred to a 25 mL PTFE reactor, heated at 120 °C for 10 h, cooled to room temperature, washed with water and ethanol more than five times, and vacuum-dried at 60 °C for 12 h to obtain NiFe-LDH/ZIF-L/NF.

### 3.2. Characterization

SEM images were obtained through an SU8020 scanning electron microscope (Hitachi, Tokyo, Japan) operating at 5.0 kV. HRTEM was obtained by transmission electron microscopy (TEM) with the F-200 model (JEOL, Akishima, Japan). Phase identification was carried out using X-ray diffraction (XRD) on a Bruker D8 Advance diffractometer (Billerica, MA, USA, Cu Kα radiation, λ = 1.54 Å), scanning from 10° to 90° (2θ). The XPS data were collected by an ESCALAB 250Xi X-ray photoelectron spectrometer (Thermo Fisher Scientific, Waltham, MA, USA). The contact angle was obtained using a contact angle measuring instrument (OCA-50). Fourier transform infrared (FTIR) spectra were acquired using a PerkinElmer Model 100 FTIR spectrometer (Waltham, MA, USA). Micro-Raman analyses were performed on a Jobin YivonLabram HR800 spectrometer (Horiba, Kyoto, Japan). The N_2_ adsorption–desorption isotherms were collected by JW-BK200C at 77 K (JWGB INSTRUMENTS, Beijing, China). Electron paramagnetic resonance (EPR) spectra were measured on a Bruker EMXPLUS spectrometer (Bruker, Berlin, Germany).

### 3.3. Electrochemical Measurements

Electrochemical measurements were conducted using the CHI660E electrochemical workstation (Chenhua Instrument, Shanghai, China) with a standard three-electrode system in 1.0 M KOH aqueous solution. The three-electrode system was made up of a working electrode (samples with an area of 1 × 1 cm^2^), reference electrode (Hg/HgO), and counter-electrode (carbon rod). All electrode potentials were converted to reversible hydrogen electrode (RHE) potentials by the Nernst equation: E vs. RHE = E vs. Hg/HgO + 0.059 pH + 0.098 V. The oxygen evolution overpotential (η) was calculated according to the following formula: η (V) = E vs. RHE—1.23 V. Linear sweep voltammetry (LSV) was performed at a scan rate of 1 mV s^−1^ from 0.8 V to 0 V. All LSV polarization curves were corrected using 90% iR compensation. The Tafel slope was plotted by converting the LSV curve according to the following formula, η = a + b log j, where η is the overpotential (mV), j is the corresponding current density (mA cm^−2^), and b is the Tafel slope (mV dec^−1^). The electrochemical surface area (ECSA) was evaluated by calculating the electrochemical double-layer capacitance (Cdl). Cdl was calculated by performing cyclic voltammetry (CV) in the non-Faradaic region (0.1–0.2 V vs. Hg/HgO) with different scan rates (v = 20–140 mV s^−1^). The Cdl was determined from the current density difference at 0.15 V vs. Hg/HgO using the equation Cdl = Ic/v, where Ic is the current density (mA cm^−2^) and v is the scan rate (mV s^−1^). Electrochemical impedance spectroscopy (EIS) measurements were carried out in the same three-electrode system with a sine wave amplitude of 10 mV, and the frequency scan range was from 10 kHz to 0.01 kHz.

### 3.4. Calculation of ECSA

The electrochemically active surface area (ECSA) was calculated according to the following equation by assuming a standard value of 60 μF/cm^2^ [50,51].

ECSA=Cdlm60 μF/cm−2

where Cdl is the specific capacitance and m is the catalyst areal loading. The nickel foam carrier does not participate in the oxygen evolution reaction, so the mass of the carrier is deducted (m_NF_ = 0.0334 mg).

## 4. Conclusions

In summary, the NiFe-LDH/ZIF-L/NF electrode with a unique sandwich-like structure exhibits excellent OER performance after reconstruction, demonstrating a low overpotential of 221 mV at 10 mA cm^−2^ and remarkable durability. The superior catalytic activity can be attributed to the following factors: (1) The composite nanosheet arrays provide a large specific surface area, exposing abundant active sites, while the hierarchical porous structure enhances mass transfer and hydrophilic gas-transport properties. (2) Self-reconstruction generates concave nanosheets enriched with oxygen vacancies, significantly increasing active site exposure and triggering the formation of NiOOH species as the true active phase for OER, thereby boosting catalytic efficiency. These findings highlight the considerable potential of MOF-derived self-supporting catalysts integrated with NiFe-LDH as cost-effective alternatives to noble metal-based electrocatalysts for alkaline OER applications.

This study mainly focuses on the structural design and electrochemical performance of the NiFe-LDH/ZIF-L/NF electrode, and has not yet explored the long-term stability of the catalyst in actual devices and the feasibility of large-scale application. Although the formation of the active phase during the self-reconstruction process has been confirmed by various characterizations, the atomic-scale details of the reaction mechanism still need to be further revealed with the help of advanced in situ characterization and theoretical calculations.

## Data Availability

All data are available in the main text or the Supplementary Materials.

## Supplementary Materials

The following supporting information can be downloaded at https://www.mdpi.com/article/10.3390/molecules30122632/s1. Figure S1: XRD pattern of ZIF-L. Figure S2: FT-IR spectra of ZIF-L/NF. Figure S3: XPS survey of (a) ZIF-L/NF, (b) NiFe-LDH/NF, and (c) NiFe-LDH/ZIF-L/NF. Figure S4: (a–c) SEM images of NiFe-LDH/ZIF-L/NF at different angles and magnifications. Figure S5: CVs of (a) ZIF-L/NF, (b) NiFe-LDH/NF, and (c) NiFe-LDH/ZIF-L/NF. Figure S6: (a) LSV curves normalized by ECSA (jECSA-noemalized) for OER and (b) Tafel slope normalized by ECSA (jECSA-noemalized) for OER. Figure S7: FT-IR spectra of NiFe-LDH/NF, NiFe-LDH/ZIF-L/NF, and NiFe-LDH/ZIF-L/NF-A. Figure S8: (a,b) SEM and (c–f) EDS images of NiFe-LDH/ZIF-L/NF-A. Figure S9: TEM image of NiFe-LDH/ZIF-L/NF-A. Table S1: The specific surface area (SBET), pore volume (Vp), and average pore size (dp) for diverse catalysts. Table S2: Cdl, areal loading (m), and ECSA of different catalysts. Table S3: The relative ratio of surface Ni2+/Ni3+ in Ni 2p XPS spectral fitting. Table S4: The relative concentration ratios of M-OH and the relative ratio of surface M-OH/M-O in O 1s XPS spectra fitting. Table S5: Summary of previously reported excellent OER catalysts. Video S1. References [42,52,53,54,55,56,57,58,59,60] are cited in the supplementary materials.
