# Supplementary material for: Rich Oxygen Vacancies Induced by Surface Self-Reconstruction in Sandwich-like Hierarchical Structured Electrocatalyst for Boosting Oxygen Evolution Reaction
Source: Molecules. 2025 Jun 17;30(12):2632. doi: 10.3390/molecules30122632 (PMC12196320; doi:10.3390/molecules30122632)
Supplement: Supplementary file 1 [file molecules-30-02632-s001.zip › Support Information.pdf]

# Support Information

# Rich oxygen vacancies induced by surface self-reconstruction in sandwich-like hierarchical structured electrocatalyst for boosting oxygen evolution reaction

Xiaoguang San[a], WanmengWu[a], XueyingLi[a], Lei Zhang[a]*, Jian Qi[bc]*, Dan Meng[a]*

[a] College of Chemical Engineering, Shenyang University of Chemical Technology, Shenyang 110142, P.R. China

[b] State Key Laboratory of Biochemical Engineering, Institute of Process Engineering, Chinese Academy of Sciences, Beijing 100190, P. R. China.

[c] School of Chemical Engineering, University of Chinese Academy of Sciences, Beijing 100049, P. R. China.

*Corresponding authors:

E-mail: laylazhang@syuct.edu.cn (L. Zhang); jqi@ipe.ac.cn (J. Qi); mengdan0610@hotmail.com (D. Meng).

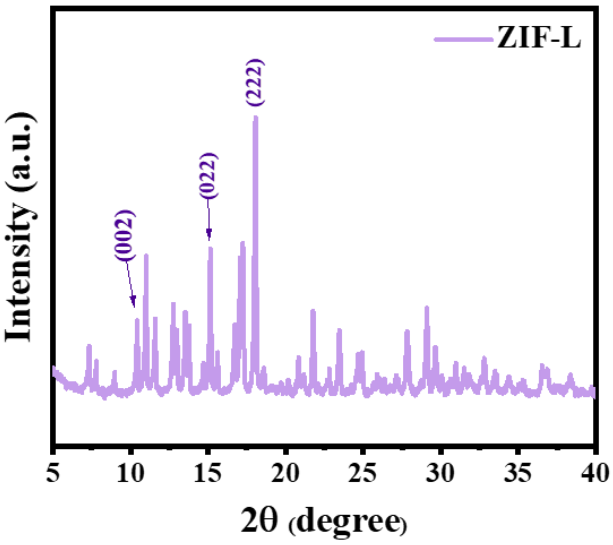


**Figure S1.** XRD pattern of the ZIF-L

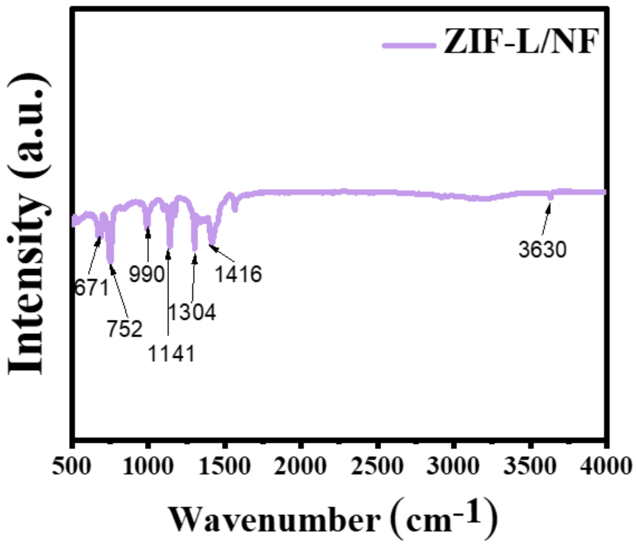


**Figure S2.** FT-IR spectra of ZIF-L/NF

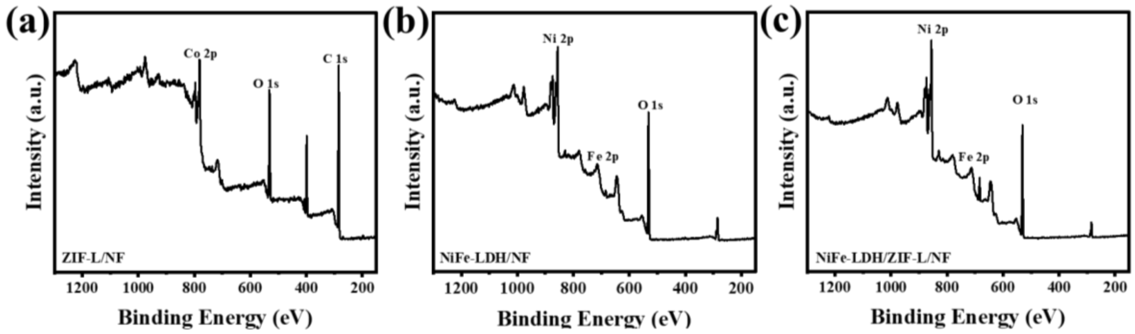


**Figure S3.** XPS survey of (a) ZIF-L/NF, (b) NiFe-LDH/NF and (c) NiFe-LDH/ZIF-L/NF.

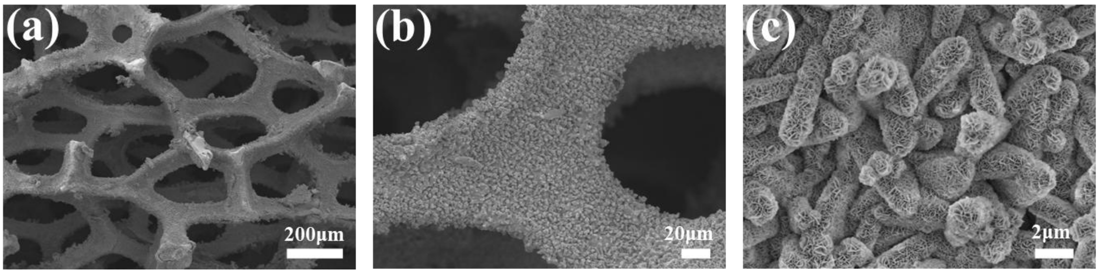


**FigureS4.** (a-c) SEM images of NiFe-LDH/ZIF-L/NF at different angles and magnifications.

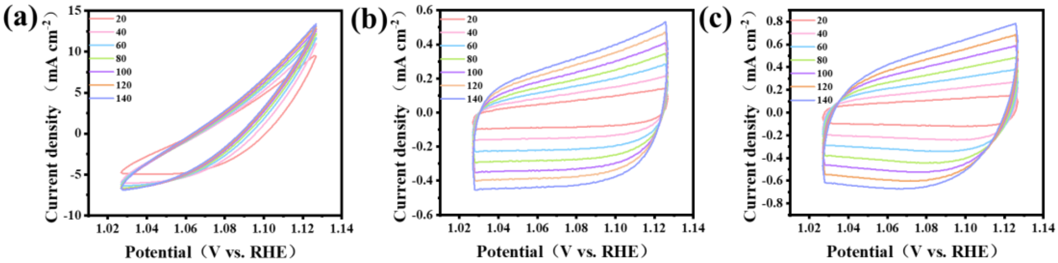


**Figure S5.** CVs of (a) ZIF-L/NF, (b) NiFe-LDH/NF and (c) NiFe-LDH/ZIF-L/NF.

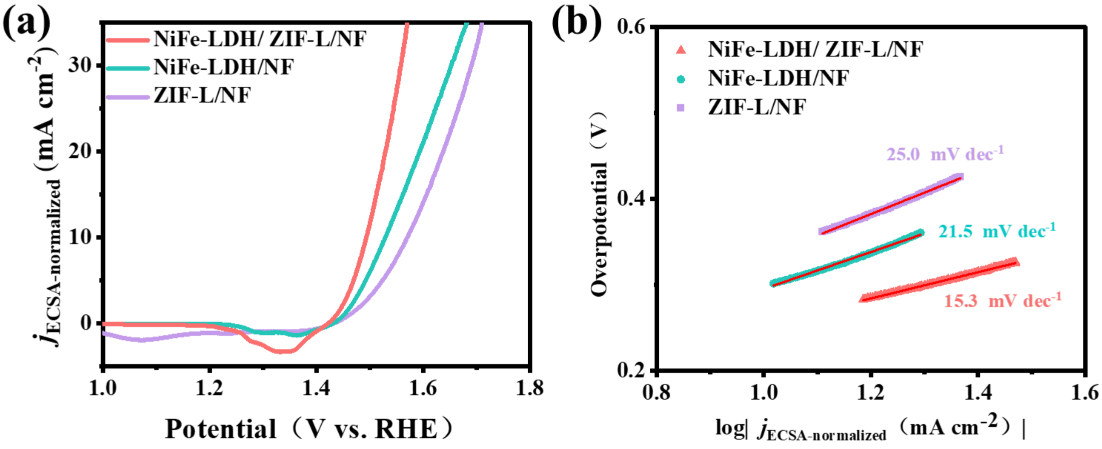


**Figure S6.** (a) LSV curves normalized by ECSA ($j_{ECSA\text{-}noemalized}$) for OER, (b) Tafel slope normalized by ECSA ($j_{ECSA\text{-}noemalized}$) for OER

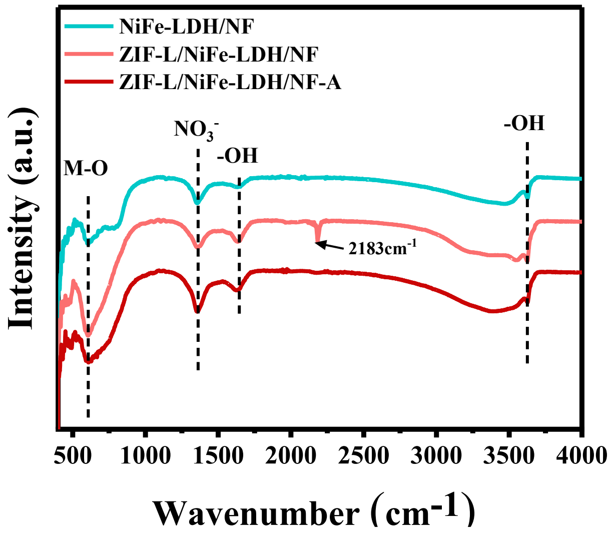


**Figure S7.** FT-IR spectra of NiFe-LDH/NF, NiFe-LDH/ZIF-L/NF and NiFe-LDH/ZIF-L/NF-A

The infrared diffraction spectrum was used to analyze the reaction. The peak at 604 $cm^{-1}$ is attributed to M-O, which confirms the presence of M-O. The peak at 1352 $cm^{-1}$ corresponds to the vibration of interlayer anions, indicating that this absorption peak corresponds to the asymmetric stretching vibration of $NO_3^-$. The absorption peaks at 3862 $cm^{-1}$ and 1643 $cm^{-1}$ are caused by the H-O stretching vibration and bending vibration, respectively, confirming the presence of M-OH. The significant O-H vibrational peak indicates that the surface is rich in polar groups, which is conducive to the adsorption of water molecules, thereby enhancing the interface interaction between the electrocatalyst and the electrolyte in OER [S1]. Furthermore, by combining the absorption peak at 3862 $cm^{-1}$ (due to hydrogen bonding) with the M-O stretching vibration peak at 604 $cm^{-1}$, the presence of M-OOH is confirmed. Compared to NiFe-LDH/NF, the absorption peaks for M-OH, M-O, and M-OOH in NiFe-LDH/ZIF-L/NF and NiFe-LDH/ZIF-L/NF-A are stronger, suggesting that the presence of ZIF-L/NF leads to a higher concentration of oxygen-containing intermediates in NiFe-LDH/ZIF-L/NF.

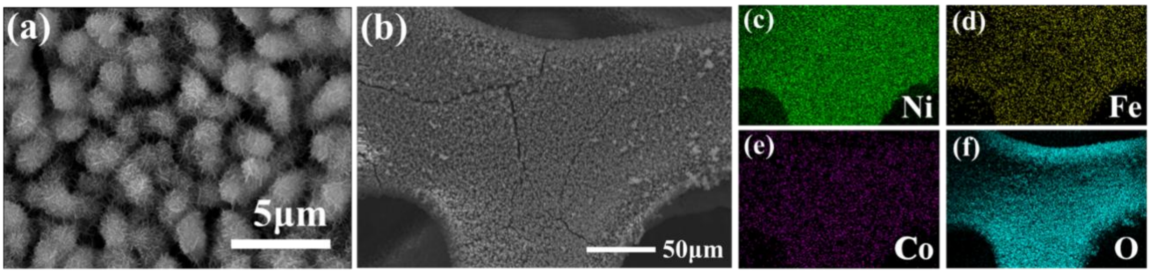


**Figure S8.** (a, b) SEM and (c-f) EDS images of NiFe-LDH/ZIF-L/NF-A.

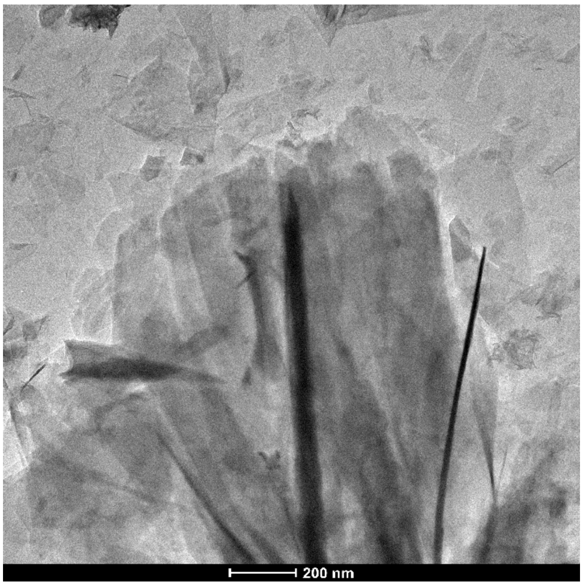


**Figure S9.** TEM image NiFe-LDH/ZIF-L/NF-A.

**Table S1.** The specific surface area ($S_{BET}$), pore volume ($V_p$), average pore size (dp) for diverse catalysts.

| Catalysts | $S_{BET}$ ($m^2g^{-1}$) | $d_p$(nm) | $V_p$($cm^3g^{-1}$) |
|---|---|---|---|
| ZIF-L/NF | 3.012 | 13.794 | 0.010 |
| NiFe-LDH/NF | 16.906 | 10.773 | 0.046 |
| NiFe-LDH/ZIF-L/NF | 35.091 | 9.294 | 0.085 |

**Table S2.** Cdl, areal loading (m) and ECSA of different catalysts

| Catalysts | ZIF-L | NiFe-LDH | NiFe-LDH/ZIF-L |
|---|---|---|---|
| m ($g/cm^{-2}$) | 0.0113 | 0.0097 | 0.0154 |
| Cdl($mF/cm^{-2}$) | 2.21 | 2.43 | 3.99 |
| ECSA ($m^2/g$) | 3.26 | 4.17 | 4.32 |

**Table S3.** The relative ratio of surface $Ni^{2+}/Ni^{3+}$ in Ni 2p XPS spectral fitting.

| Catalysts | $Ni^{2+}/Ni^{3+}$ |
|---|---|
| NiFe-LDH/NF | 1.12 |
| NiFe-LDH/ZIF-L/NF | 1.25 |
| NiFe-LDH/ZIF-L/NF-A | 0.62 |

**Table S4.** The relative concentration ratiosof M-OH and the relative ratio of surface M-OH/M-O in O 1s XPS spectra fitting.

| Catalysts | M-OH/M-O |
|---|---|
| ZIF-L/NF | 9.12 |
| NiFe-LDH/NF | 16.12 |
| NiFe-LDH/ZIF-L/NF | 27.90 |
| NiFe-LDH/ZIF-L/NF-A | 35.37 |

**Tabel S5.** Summary of previously reported excellent OER catalysts.

| Catalysts | Load | Electrolyte | $\eta_{10}$ (mV) | Ref. |
|---|---|---|---|---|
| NiFe-LDH/ZIF-L/NF | NF | 1M KOH | 221 | This work |
| (Co,Ni)$Se_2$@NiFe LDH | - | 1M KOH | 277 | [S2] |
| RuSe2@NiFeLDH | - | 1M KOH | 268 | [S3] |
| NiCoLDH@NiFe-MIL | NF | 1M KOH | 270 | [S4] |
| CoO–$Co_4N$@NiFe-LDH | NF | 1M KOH | 231 | [S5] |
| Ru@CoFe-LDH | NF | 1M KOH | 249 | [S6] |
| Fe-Ni LDH/MOF-b2 | - | 1M KOH | 255 | [S7] |
| CoFe LDH/MOF-0.06 | CC | 1M KOH | 276 | [S8] |
| ZIF-67/CoNiAl-LDH | NF | 1M KOH | 303 | [S9] |
| Ce-CoFe-LDH/NF | NF | 1M KOH | 225 | [S10] |

## References

[S1] Guan, D.; Xu, H.; Huang, Y. C.; Jing, C.; Tsujimoto, Y.; Xu, X.; Lin, Z.; Tang, J.; Wang, Z.; Sun, X., Operando studies redirect spatiotemporal restructuration of model coordinated oxides in electrochemical oxidation. *Advanced Materials***2025**, 37, 2413073.

[S2] Li, J.-G.; Sun, H.; Lv, L.; Li, Z.; Ao, X.; Xu, C.; Li, Y.; Wang, C., Metal–organic framework-derived hierarchical (Co,Ni)$Se_2$@NiFe LDH hollow nanocages for enhanced oxygen evolution. *ACS applied materials & interfaces* **2019,** 11, 8106-8114.

[S3] Kumar, S.; Raju, S.; Marappa, S.; S, M.; DR, V., Unlocking the Potential of

Water Splitting: FeMn-LDH/$MoS_2$ Composite with Enhanced Activity and Durability. *ACS Applied Energy Materials* **2024,** 7, 9872-9881.

[S4] Yang, L.; Jin, L.; Wang, K.; Xu, H.; He, G.; Chen, H., Interface coupling induced built-in electric fields boost electrocatalytic oxygen evolution reaction over MOF@ LDHs core-shell nanocones. *Colloids and Surfaces A: Physicochemical and Engineering Aspects* **2023,** 672, 131720.

[S5] Chen, B.; Humayun, M.; Li, Y.; Zhang, H.; Sun, H.; Wu, Y.; Wang, C., Constructing hierarchical fluffy CoO–$Co_4N$@ NiFe-LDH nanorod arrays for highly effective overall water splitting and urea electrolysis. *ACS Sustainable Chemistry & Engineering* **2021,** 9, 14180-14192.

[S6] Karmakar, A.; Jayan, R.; Das, A.; Kalloorkal, A.; Islam, M. M.; Kundu, S., Regulating surface charge by embedding Ru nanoparticles over 2D hydroxides toward water oxidation. *ACS Applied Materials & Interfaces* **2023,** 15, 26928-26938.

[S7] Huo, J.; Wang, Y.; Yan, L.; Xue, Y.; Li, S.; Hu, M.; Jiang, Y.; Zhai, Q.-G., In situ semi-transformation from heterometallic MOFs to Fe–Ni LDH/MOF hierarchical architectures for boosted oxygen evolution reaction. *Nanoscale* **2020,** 12, 14514-14523.

[S8] Huang, S.; Wu, Y.; Fu, J.; Xin, P.; Zhang, Q.; Jin, Z.; Zhang, J.; Hu, Z.; Chen, Z., Hierarchical CoFe LDH/MOF nanorods array with strong coupling effect grown on carbon cloth enables efficient oxidation of water and urea. *Nanotechnology* **2021,** 32, 385405.

[S9] Xu, J.; Zhao, Y.; Li, M.; Fan, G.; Yang, L.; Li, F., A strong coupled 2D metal-organic framework and ternary layered double hydroxide hierarchical nanocomposite as an excellent electrocatalyst for the oxygen evolution reaction. *Electrochimica Acta* **2019,** 307, 275-284.

[S10] Wang, L.; Liu, Y.; Liu, X.; Chen, W., 3D nanostructured Ce-doped CoFe-LDH/NF self-supported catalyst for high-performance OER. *Dalton Transactions* **2023,** 52, 12038-12048.
