# Supplementary material for: Rich Oxygen Vacancies Induced by Surface Self-Reconstruction in Sandwich-like Hierarchical Structured Electrocatalyst for Boosting Oxygen Evolution Reaction
Source: Molecules. 2025 Jun 17;30(12):2632. doi: 10.3390/molecules30122632 (PMC12196320; doi:10.3390/molecules30122632)
Supplement: Supplementary file 1 [file molecules-30-02632-s001.zip › Video Abstract.pdf]

Video abstract

This study successfully synthesized a "sandwich"-structured NiFe-LDH/ZIF-L/NF catalyst via a combination of room-temperature immersion and hydrothermal treatment. In the accompanying video demonstration, when a droplet contacts the material's surface, it rapidly spreads and vanishes instantaneously. Dynamic contact angle measurements confirm its superhydrophilic nature, attributed to the material's porous architecture and the presence of hydrophilic functional groups. This exceptional hydrophilicity facilitates full contact between the catalyst and the electrolyte, significantly enhancing mass transfer efficiency during the oxygen evolution reaction (OER) process.
